# Aligning Practice With Guidelines: A Canadian National Survey and Canadian Society of Nephrology Commentary on the 2025 KDIGO Pediatric Nephrotic Syndrome Recommendations

**DOI:** 10.1177/20543581261455537

**Published:** 2026-05-28

**Authors:** Seetha Radhakrishnan, Allison B. Dart, Mallory L Downie, Silviu Grisaru, Anna Mathew, Reem A. Mustafa, Cherry Mammen

**Affiliations:** 1 Division of Pediatric Nephrology, 7979 The Hospital for Sick Children, Toronto, ON; 2 Division of Pediatric Nephrology, Children’s Hospital, Health Sciences Centre, Children’s Hospital Research Institute of Manitoba, 8664 University of Manitoba Winnipeg, MB; 3 Division of Pediatric Nephrology, Montreal Children’s Hospital, 507266 Research Institute of the McGill University Health Centre, Montreal, QC; 4 Division of Pediatric Nephrology, Alberta Children's Hospital, Calgary, AB; 5 Division of Nephrology, St Joseph’s Hospital, 3710 McMaster University, Hamilton, ON; 6 Devision of Nephrology and Hypertension and the Evidence-based Practice and Impact Center, Department of Internal Medicine, 21638 University of Kansas Medical Center, Kansas City, KS, USA; 7 Division of Pediatric Nephrology, 8166 British Columbia Children’s Hospital, Vancouver, BC

**Keywords:** childhood nephrotic syndrome, clinical practice guidelines, glomerular disease, pediatric nephrology, canadian

## Abstract

**Purpose of Review:**

The purpose of this commentary is to review the 2025 Kidney Disease Improving Global Outcomes (KDIGO) clinical practice guidelines on the management of childhood nephrotic syndrome (NS) within the Canadian context and based on current practices across the country.

**Sources of information:**

The KDIGO 2025 guideline on the management of childhood nephrotic syndrome as well as the KDIGO 2021 management of glomerular diseases, chapter 4 (NS in children) were main sources. The International Pediatric Nephrology Association clinical practice recommendations for the diagnosis and management of children with steroid sensitive NS and the recommendations for the diagnosis and management of children with steroid resistant NS were also used.

**Methods:**

The Canadian Society of Nephrology convened a working group of pediatric nephrologists with representation from across Canada. As part of the review, the working group conducted a survey of pediatric nephrology centres in Canada to better understand current practice in NS management. All members reviewed the recommendations and practice points, reaching consensus through discussion. Results from the survey as well as published literature from Canadian NS cohorts were used to develop commentary on the application of KDIGO recommendations in the Canadian context.

**Key Findings:**

The authors agreed with most updated KDIGO recommendations and emphasized the benefits of standardizing disease and response type definitions, as well as prednisone induction protocols. However, areas in need of further clarity and standardization were highlighted including relapse treatment protocols, and choice of steroid sparing agents for frequently relapsing and steroid dependent patients.

**Limitations:**

A review of the quality of evidence was not undertaken.

## Abstract (fr)

**Motif de la revue:**

Ce commentaire passe en revue les lignes directrices KDIGO 2025 sur la prise en charge du syndrome néphrotique (SN) de l’enfant dans le contexte canadien et selon les pratiques ayant cours à travers le pays.

**Sources de l’information:**

Les principales sources utilisées étaient les lignes directrices KDIGO 2025 sur la prise en charge du syndrome néphrotique de l’enfant et le chapitre 4 des lignes directrices KDIGO 2021 sur les maladies glomérulaires (SN chez l’enfant). Les recommandations de pratique clinique de l’International Pediatric Nephrology Association pour le diagnostic et la prise en charge du syndrome néphrotique corticodépendant et corticorésistant chez l’enfant ont également été prises en compte.

**Méthodologie:**

La Société canadienne de néphrologie a constitué un groupe de travail composé de néphrologues pédiatriques de partout au Canada. Le groupe de travail a réalisé une enquête auprès des centres canadiens de néphrologie pédiatrique afin de mieux caractériser les pratiques actuelles de prise en charge du SN. Tous les membres ont révisé les recommandations et les points de pratique, et sont parvenus à un consensus après discussion. Les résultats de l’enquête, ainsi que les données publiées de cohortes canadiennes de SN ont servi à élaborer un commentaire sur l’application des recommandations du KDIGO dans le contexte canadien.

**Principaux résultats:**

Les auteurs appuyaient la majorité des recommandations du KDIGO mises à jour et ont souligné les avantages de standardiser les définitions de la maladie et des types de réponse, ainsi que les protocoles d’induction par prednisone. Cependant, des besoins de clarification et de standardisation sont demeurés, notamment pour les protocoles de traitement des rechutes et le choix des agents d’épargne stéroïdienne chez les patients à rechutes fréquentes ou corticodépendants.

**Limites:**

La qualité des données probantes n’a pas été évaluée.

## Introduction

Idiopathic nephrotic syndrome (NS) is the most frequent glomerular disease in children; however its etiology and pathophysiology remain elusive and individual disease trajectories are difficult to predict at presentation.^1^ Most affected children achieve complete remission of proteinuria with daily corticosteroids within 4 weeks, but 70–80% will experience at least one relapse and 40-50% will have frequent relapses.^2,3^ As such, a large proportion of children will be exposed to corticosteroids repeatedly and will have an increased risk for steroid toxicity. This has led to an ongoing debate as to the optimal dose and duration of corticosteroid treatment for NS, as well as optimal timing and selection of steroid sparing agents.^2-4^

Care of childhood NS in Canada is primarily delivered by pediatric nephrologists at 13 tertiary care centres across seven provinces with separate health authorities. There exists considerable practice variation across the centres, especially in regards to the use of steroid sparing agents for frequently relapsing nephrotic syndrome (FRNS) and steroid dependent (SDNS) patients.^5^ Canadian epidemiology, outcomes, and management practices have been shared through two well established nephrotic syndrome cohorts: The Canadian Childhood Nephrotic Syndrome Project (CHILDNEPH) and Investigating Genes, Health and Therapeutics (INSIGHT).^6,7^ CHILDNEPH is a national observational study of 328 children with steroid-sensitive NS (SSNS) recruited and observed across 11 Canadian sites from 2013 to 2019. INSIGHT is a prospective childhood NS cohort of over 800 children recruited since 2011 from the Greater Toronto and Hamilton Area and has principally been run at The Hospital for Sick Children.

Clinical practice guidelines help standardize care and reduce variability while promoting evidence-based medicine towards improving patients’ outcomes and safety. In 2012, the Kidney Disease: Improving Global Outcomes (KDIGO) initiative provided one of the first sets of guidelines addressing glomerulonephritis, including a chapter covering childhood NS.^8,9^ Recognizing the fact that regional factors warrant consideration when applying clinical practice guidelines to specific populations, the Canadian Society of Nephrology (CSN) and representatives from the Canadian Association of Pediatric Nephrologists published a commentary on the relevancy and applicability of these guidelines in the Canadian context in 2013.^10^

In response to a growing body of new evidence, KDIGO released updated guidelines in 2021 on the management of glomerulonephritis, including childhood NS (KDIGO 2021).^11^ Additionally, KDIGO published a 2025 Clinical Practice Guideline for the Management of Nephrotic Syndrome in Children (KDIGO 2025), a focused update in pediatrics, reflecting new randomized controlled trial evidence through August 2024.^12^ The update emphasized shorter corticosteroid regimens for initial therapy, clarified definitions, discouraged routine prophylactic steroids during infections, and provided expanded guidance on glucocorticoid-sparing therapies.^12^ Pediatric NS guidelines have also been published in 2020 and 2022, by the International Pediatric Nephrology Association (IPNA) on the diagnosis and treatment of children with steroid resistant and steroid sensitive nephrotic syndrome respectively.^2,13^

These new guidelines incorporate contemporary research findings, novel treatment options, and practical recommendations requiring careful consideration when applied to the Canadian healthcare context. As such, the CSN has again assembled a working group to review the newly published KDIGO guidelines. To assess implementation and impact on practice, the working group also developed a survey that was distributed to pediatric nephrologists in Canada. We share below the results of this survey along with a commentary focusing on application of the KDIGO 2025 guidelines within Canada and offer insights drawn from Canadian clinical practice and recent national research.

## Process and Structure

The CSN Clinical Practice Guideline Committee (CSN CPGC) has previously developed standard methods for reviewing existing KDIGO guidelines.^14^ These methods incorporate how to review and appraise graded recommendations and practice points. Graded recommendations are supported by systematic reviews of the evidence; however, practice points are typically not. Practice points are intended to guide the implementation of graded recommendations, offer “good practice” statements that are based on compelling indirect evidence, or include ungraded statements based on testable questions that were not subject to a formal systematic review.

The main working group consisted of a Chair and co-Chair, appointed by the CSN CPGC. The chairs appointed a separate working group of pediatric nephrologists through invitations to CSN members with known expertise in the topic. The pediatric group was tasked initially with the review of chapter 4, Nephrotic Syndrome in Children, from KDIGO 2021 and subsequently the updated KDIGO 2025 supplement, in order to develop a commentary specifically devoted to the review of pediatric nephrotic syndrome.^11,12^

The pediatric nephrology working group, herein referenced as the working group, included 5 pediatric nephrologists from across Canada. Formed in 2024, the working group met virtually to review all recommendations and practice points in the guideline. As part of this review, recent clinical practice recommendations from IPNA and published literature were taken into consideration.^2,13^ To offer further Canadian context, data from national research in childhood NS from both the INSIGHT and CHILDNEPH cohorts has been incorporated.

In order to explore the Canadian implementation of the KDIGO guidelines and areas for future development, a survey was created (Appendix A) by the working group. This survey was completed through emails and interviews with a representative from each Pediatric Nephrology centre across the country in November 2024, prior to publication of the KDIGO 2025 guidelines. Questions were developed to assess centre specific practice in relation to prednisone dose and duration for initial episode and relapses, choice and funding of steroid sparing medications, and diagnosis/treatment of steroid resistant patients. Results were collated and have been included in relevant sections of the commentary. Consensus was achieved by the working group on all provided recommendations, and all authors have approved the final text. The CSN CPGC arranged peer review of the commentary, following which the working group undertook revisions based on reviewer comments.

The structure of this paper includes a review of KDIGO 2025 recommendations followed by subsections on topic areas within pediatric NS where commentary is further provided. Table 1 includes a list of all recommendations and select practice points, along with a consensus statement by the working group. Each recommendation and practice point from KDIGO 2025 was reviewed; however only practice points that the working group felt were high yield for clinical practice are discussed. Recommendations that have changed from KDIGO 2021 are bolded. Within each subsection, relevant KIDGO recommendations and practice points are presented, commentary is provided incorporating recent literature and international guidelines, implications of these recommendations and practice points within Canadian Healthcare are explored, and suggestions are made on future directions for research.

**Table 1.** Select KDIGO Recommendation Statements and Practice Points on Management of Childhood Nephrotic Syndrome Adapted From KDIGO 2025. Recommendations That Have Changed From KDIGO 2021 Are Bolded

No.	Recommendation statement and practice points	Concur
**1.1.1.**	Practice Point	See comment
	**The definitions relating to nephrotic syndrome in children are based on the clinical characteristics as defined in the KDIGO 2025 guideline** ^12^ **.**	
	Initial Treatment of nephrotic syndrome	
1.3.1.1	Recommendation	Yes
	We recommend that oral glucocorticoids be given for 8 weeks (4 weeks of daily glucocorticoids followed by 4 weeks of alternate-day glucocorticoids) or 12 weeks (6 weeks of daily glucocorticoids followed by 6 weeks of alternate-day glucocorticoids) (1B)	
**1.3.1.1**	Practice Point	See comment
	The standard dosing regimen for the initial treatment of nephrotic syndrome is daily oral prednisone/prednisolone 60 mg/m2/d or 2 mg/kg/d (maximum 60 mg/d) for 4 weeks followed by alternate day prednisone/prednisolone, 40 mg/m2, or 1.5 mg/kg (**maximum of 40 mg)** for other 4 weeks, or prednisone/prednisolone 60 mg/m2/d (maximum 60 mg/d) for 6 weeks followed by alternate day prednisone/prednisolone, 40 mg/m2, or 1.5 mg/kg (**maximum of 40 mg**), for other 6 weeks.	
	Prevention and Treatment of Relapses	
**1.3.2.1**	Recommendation	Yes
	**For children with frequently relapsing and steroid-dependent nephrotic syndrome we recommend that daily glucocorticoids not be routinely given during episodes of upper respiratory tract and other infections to reduce the risk of relapse (1C).**	
**1.3.2.1**	Practice Point	Yes
	**A short course (i.e., 3 extra doses) of low-dose (0.5 mg/kg per day), daily prednisone or prednisolone at the onset of an upper respiratory tract infection can be considered in children with frequently relapsing and steroid-dependent nephrotic syndrome who are already taking low-dose, alternate-day prednisolone and have a history of repeated infection-associated relapses or significant prednisone- or prednisolone-related morbidity.**	
1.3.3.1	Practice Point	Yes
	The initial approach to relapse should include oral prednisone/prednisolone as a single daily dose of 60 mg/m2/d or 2 mg/kg/d (maximum 60 mg/d) until the child remits completely for >/=3 days	
1.3.3.2	Practice Point	Yes
	After achieving complete remission, reduce oral prednisone/prednisolone to 40 mg/m2 or 1.5 mg/kg (**maximum 40 mg**) on alternate days for **4 weeks**.	
**1.3.3.3**.	Practice Point	Yes
	For children with frequently relapsing nephrotic syndrome or steroid-dependent nephrotic syndrome without glucocorticoid toxicity, the same glucocorticoid regimen may be employed in subsequent relapses while a **shorter taper and/or more robust steroid-sparing approaches should be considered in children with signs of glucocorticoid toxicity**.	
1.3.3.4	Practice Point	See comment
	For children with frequently relapsing nephrotic syndrome without serious glucocorticoid-related adverse effects, low-dose alternate-day oral prednisone/prednisolone (optimally</= 0.5 mg/kg/d) can be prescribed to prevent relapse	
1.3.3.1	Recommendation	See comment
	For children with frequently relapsing nephrotic syndrome who develop serious glucocorticoid-related adverse effects and for all children with steroid-dependent nephrotic syndrome, we recommend that glucocorticoid-sparing agents be prescribed, rather than no treatment or continuation with glucocorticoid treatment alone (1B)	
1.3.3.6	Practice Point	See comment
	Choosing the most appropriate glucocorticoid-sparing agent from among oral CNIs, cyclophosphamide, levamisole, MMF, and rituximab **(listed here in an unbiased order)** is a decision that requires careful consideration of specific patient-related issues such as resources, adherence, adverse effects, and patient preferences. Oral cyclophosphamide and levamisole may be preferable glucocorticoid-sparing therapies in frequently relapsing nephrotic syndrome. MMF, rituximab, CNIs, and, to a lesser extent, oral cyclophosphamide may be preferable glucocorticoid-sparing therapies in children with steroid-dependent nephrotic syndrome	
	Treatment of Steroid Resistant Nephrotic Syndrome	
1.4.4.1	Recommendation	Yes
	We recommend using cyclosporine or tacrolimus as initial second-line therapy for children with steroid-resistant nephrotic syndrome (1C)	

Bolded statements include recommendations that have changed from KDIGO 2021.

### Definitions Relating to Nephrotic Syndrome

#### Practice Point 1.1.1

##### Commentary

The KDIGO 2025 guidelines include a list of definitions relating to childhood NS, which is revised compared to KDIGO 2021, and is now consistent with IPNA definitions. Overall, the working group is pleased to see more international uniformity with specific definitions as it allows for proper comparison of data across sites, consistent implementation of guidelines, and harmonization for future multi-centre research including randomized controlled trials (RCT).^15^

The main definition update in KDIGO 2025 was the change in definition of FRNS. KDIGO 2025 now defines FRNS as ≥ 2 relapses in the first 6 months following initial remission or ≥ 3 relapses in any subsequent 12 month period, which is in alignment with IPNA. KDIGO 2021 defined FRNS as ≥ 2 relapses within 6 months of disease onset or ≥ 4 relapses in any 12 month period. The use of a uniform definition for FRNS is important considering this is one of the main indications for the use of steroid sparing medications. An INSIGHT study revealed that among 1054 Canadian children with SSNS, 27% would be classified with FRNS based on KDIGO 2021 criteria vs. 36% by KDIGO 2025 or IPNA criteria.^16^ Implementation of the 2025 KDIGO & IPNA FRNS criteria would potentially facilitate earlier steroid-sparing medication initiation to prevent relapses and steroid toxicity.

### Initial Treatment of NS in Children

#### Recommendation 1.3.1.1 and Practice Point 1.3.1.1

##### Commentary

Based on strong RCT evidence and a Cochrane systematic review published since the 2012 KDIGO guidelines, KDIGO 2025 maintains the recommendations to limit initial corticosteroid exposure to 8-12 weeks.^3,17-19^ Overall, recent trials showed no significant benefit in prolonging treatment past 12 weeks with respect to delaying time to first relapse and reducing relapse frequency. This recommendation should lead to reduced practice variation in prednisone dosing amongst pediatric nephrologists, which was significant from historical surveys.^20^ Even though studies did not observe significantly increased prednisone side effects with longer courses, the goal of providers is to limit corticosteroid exposure as much as safely possible considering most children with NS will have a chronic relapsing phenotype. As such, future research should continue to explore further reduction in dose and duration of induction prednisone, maximum dosing for larger children, as well as comparisons of body surface area (BSA) versus weight-based dosing. The recommended maximum dosing of daily prednisone is the same between KDIGO 2025 and IPNA and is now also the same for maximum alternate day prednisone dosing of 40mg. This is an update from the KDIGO 2021, which stated a maximum of 50 mg alternate day. The working group is supportive of this change, given that higher dosage of corticosteroids has not been shown to provide significant benefit.^3^ In regards to weight versus BSA based dosing, a prior study has shown that in patients <30 kg, a regimen using 2 mg/kg/day provided significantly less prednisone compared to a course using 60 mg/m^2^/day.^21^ In a randomized trial comparing body weight versus BSA dosing in children, cumulative prednisolone dose at 6 months and incidence of hypertension was higher in the BSA dosing arm, but overall relapse rates were similar between both arms.^22^

##### Implications Within Canadian Healthcare

Data from CHILDNEPH revealed that practice variation of prednisone dose and duration still exists and is mostly explained at the site level compared to individual physician and patient factors.^5^ In this study, induction prednisone therapy was more similar across sites compared to relapse treatments. In our survey for this commentary, 11/12 (92%) Canadian pediatric nephrology sites use standardized induction protocols for prednisone dose and duration following diagnosis. Three sites are using slightly increased duration of tapering prednisone compared to KDIGO recommendations. Centres were evenly split between mg/kg and mg/m^2^ dosing and duration of initial daily dosing between 4 versus 6 weeks. All but one centre limited maximum induction steroid dose to 60mg daily; however, maximum dose for tapering prednisone was variable. Half of the centres used a maximum dose of 40 mg every other day, while others used 50 or 60 mg. Variability in maximum dose for tapering prednisone is likely reflective of the discrepancy in previous guidelines as discussed above.^11,12^ The working group would like to see nationwide consensus on limiting initial corticosteroid exposure to 8-12 weeks, limiting induction dose to a maximum of 60mg, and standardizing tapering dose and regimens further, given that longer duration and higher dosage of exposure does not provide significant benefit.^3,18,19^

### Prevention of Relapses During Intercurrent Illness

#### Recommendation 1.3.2.1 and Practice Point 1.3.2.1

##### Commentary

It is well established that infections are the most common triggers for NS relapses in children. Based on results from 4 RCT’s, KDIGO 2021 recommended prescribing pre-emptive daily glucocorticoids during infection in order to minimize the risk of relapse. These initial studies involved smaller sample sizes and were at high risk of bias due to either cross-over or open label study designs.^23-26^

Since publication of the 2021 guideline, the PREDNOS 2 study, a well-designed RCT including a multi-ethnic cohort of 365 patients from the United Kingdom, showed no benefit of this strategy in preventing relapses.^27^ Patients were randomized to a treatment arm consisting of prednisolone 15 mg/m2 daily or placebo for a duration of 6 days at the onset of an URTI. The percentage of patients experiencing a URTI related relapse was similar in both treatment arms. Post-hoc analysis also did not find that background ethnicity or concomitant immunosuppression had any influence on the outcome. With the results from this trial, KDIGO 2025 has changed its recommendation, stating that glucocorticoids should not be given routinely during infections to decrease risk of relapse. The working group supports this change in recommendations as it is based on current evidence and now aligns with recommendations from IPNA and a separate KDOQI commentary.^2,28^ Furthermore, the practice point 1.3.2.1, aligns with the IPNA guidelines suggesting a short course of low dose daily prednisone at the onset of a URTI can be considered in those already taking low dose alternate prednisone, having a history of repeated infection-associated relapses, or steroid related side effects.

##### Implications Within Canadian Healthcare

From our Canadian survey, while there is some practice variation around this topic, we note that many are already in alignment with the updated 2025 KDIGO recommendations. When asked if low daily doses of prednisone are being prescribed for infections in order to prevent a relapse, half of the sites stated “Never” or “Rarely”, 4/12 (25%) sites stated “Sometimes” while only 2/12 (17%) said “Always”. In all sites that follow this practice, they mention prescribing small daily prednisone doses only with patients already on alternate day prednisone.

### Treatment of Relapses

#### Practice Point 1.3.3.1 and Practice Point 1.3.3.2

##### Commentary

The working group agrees with the initial approach of treating relapses as highlighted in Practice Point 1.3.3.1. However, the optimal prednisone dose and duration for NS relapses is not as evidence-based compared to the induction course. Only 2 RCT’s have been completed since the 2012 KDIGO GN guideline investigating duration of prednisone tapering. Overall a dose of 40 mg/m^2^ every other day for 4 weeks subsequent to induction of remission was supported in these trials,^29,30^ but the question of whether lower prednisone doses and duration could be used to effectively treat relapses is still unknown. A recent non-inferiority single centre RCT from India found that a “short” regimen of 40 mg/m^2^ on alternate days for 2 weeks after remission versus a “standard” 4 weeks resulted in a similar proportion of patients developing FRNS and SDNS.^29^ Time to relapse and overall relapse rates were also similar in both groups. One major issue of the study was that it could not establish non-inferiority based on the primary outcome, likely due to small sample size. Therefore, further well-designed research studies on relapse treatment are necessary to determine the minimal corticosteroid exposure necessary for standard relapses. Yet to be published results from trials such as the RESTERN study in the Netherlands may shed more light on this topic.^31^ Future studies on relapse dosing will be especially important for children of younger age (<4 years), who are more prone to frequent relapses.

The working group would also like to highlight the slight variation between KDIGO 2025 and 2021 in relapse dosing after remission. KDIGO 2025 suggests an alternate day prednisone dose at 40 mg/m2 (or 1.5 mg/kg) for exactly 4 weeks with a maximum dose of 40 mg versus the KDIGO 2021 recommendation of the same dose for ≥4 weeks with a maximum dose of 50 mg. IPNA’s recommendation surrounding relapse treatment is more consistent with KDIGO 2025. The reasoning for the KDIGO 2021 recommendation of a maximum dose of 50 mg is unclear, especially considering the 2012 guidelines also suggested a maximum of 40 mg for relapse alternate day dosing.

##### Implications Within Canadian Healthcare

Within Canada, there is practice variability in the course and duration of corticosteroids in the treatment of NS relapse, perhaps reflective of variation in international guidelines. Our Canadian survey results reveal that 11/12 (92%) sites have a prednisone treatment protocol for standard relapse. After achieving remission, there was wide variation in the alternate day prednisone course. 4/12 (33%) sites utilize 4 weeks of alternate day prednisone at either 40 mg/m2 or 1.5 mg/kg per dose. 3/12 (25%) sites use the same approach, but alter the number of weeks depending on the risk profile of the patient (*e*.*g*.: older vs younger, frequently vs infrequently relapsing). Other approaches included 40mg/m2 alternate day for 2 weeks, an alternate day tapering schedule over 4 weeks, and a daily prednisone wean over 4 weeks. It is clear that further evidence is needed to guide physicians on the optimal dose and duration of corticosteroid therapy to effectively treat relapses and minimize risk of cumulative steroid toxicity. A clinical trial on the effectiveness of reduced dose treatment, Reduced-dose Steroid Protocol for Childhood Nephrotic Syndrome (RESPONSE), is being planned in Canada, with a pilot study currently underway (clinical trial NCT06635720).

### Use of Corticosteroid-Sparing Agents to Treat FRNS and SDNS

#### Recommendation 1.3.3.1, Practice Point 1.3.3.4 and 1.3.3.6

##### Commentary

Approximately 50% of children with NS will experience frequent relapses or become steroid dependent despite adequate induction and relapse therapy.^2^ Repeated courses of corticosteroids put children at higher risk for corticosteroid toxicity. KDIGO 2025 recommends that FRNS patients with serious corticosteroid side effects and all patients with SDNS should start a second-line agent. The IPNA guidelines more strongly recommend maintenance treatment for all patients with FRNS or SDNS with the following criteria: not controlled on therapy, have suffered a complicated relapse, or with SDNS.^2^ The working group agrees with the overall strategy surrounding the use of steroid sparing agents and supports the use of these medications before the onset of steroid adverse effects with potential long-term consequences. A long duration of observation time is often needed for certain steroid side effects to manifest including poor growth and bone disease.^32^

The largest area of uncertainty in the management of NS remains in the selection of second line agents for FRNS and SDNS. Some changes had been made in KDIGO 2025 and 2021, including removal of chlorambucil as a recommended option and further RCT evidence supporting the use of Rituximab.^33,34^ Rituximab is recommended by the IPNA guidelines for children not controlled on therapy after a course of treatment of one of the other second line agents. The working group agrees with this recommendation.

An ongoing issue is the lack of head-to-head trials of the many available treatment options in improving relapsing outcomes. To date, there has not been a study showing superiority of one steroid sparing agent over another. For this reason, the second line agents are now listed in an unbiased order in KDIGO 2025, an approach which is supported by this group. In an emulated RCT among INSIGHT participants, there was no significant difference in relapse rates, kidney function, or subsequent steroid-sparing medication use after calcineurin inhibitor (CNI) vs. cyclophosphamide initiation. However, children treated with CNI were more likely to be hospitalized and receive intravenous albumin during relapses, which may relate to its potential nephrotoxicity.^35^ A recent CHILDNEPH study also showed no statistically significant difference in time to first relapse with initial steroid sparing therapies including cyclophosphamide, tacrolimus, mycophenolate, cyclosporine and rituximab.^5^

The selection of steroid sparing agents for SSNS should therefore be based on shared decision making between physician and family, based on preferred duration of treatment and comparative side effect profile. An important consideration is cost and access to coverage in individual provinces, especially for families without third party insurance. The working group agrees that multiple patient related variables must be considered in terms of steroid sparing agent selection including side effects, tolerability, and adherence in addition to drug cost and access. Additional considerations are the higher risk of relapses in younger children, and the relatively higher dose/kg versus BSA in this population, which may warrant earlier initiation of a second line agent.^28^

##### Implications for the Canadian Healthcare System

Due to the lack of clear evidence, there has been significant practice variation in the use of steroid sparing agents for patients with FRNS/SDNS in Canada.^10^ A CHILDNEPH publication looking at practice variation in the use of initial steroid sparing agents in Canada between 2013-2019, confirmed the wide variation in choice and timing of agents across the country. Cyclophosphamide and tacrolimus were found to be the most frequently used initial therapies at 39% and 23% respectively.^5^ The more recent working group survey found only 1 pediatric centre in Canada currently has a protocol for second line agent initiation. There was identified variability in the first choice of second line agent at each centre; cyclophosphamide (n=4), tacrolimus (n=4), mycophenolate (n=3), or cyclosporine (n=1). Rituximab is only used as a third, fourth or fifth line agent across all sites. Levamisole is not available in any province despite positive endorsement by some providers. There was additional variability in second or third choice of agent without a clear pattern. While tacrolimus was endorsed within the top three choices at all centres, cyclophosphamide was only utilized as a fourth or fifth option in 5 centres. The working group would like to see future engagement in RCTs in NS across the nation to investigate the efficacy of second line agents in FRNS/SDNS to guide management.

While rationale for current choices was not captured by the survey, there were differences in access to funding identified in each province. Notably, only British Columbia has funding through a special renal program for all immunosuppressants for eligible patients. Most provinces have provincial coverage for mycophenolate, cyclosporine, and cyclophosphamide if private insurance is not available. Notably, tacrolimus is only available via private insurance in some provinces. The differences in cost and funding models are therefore likely a driver of variation in practice across the nation. Access to Rituximab is universally more challenging. There is provincial funding in a minority of centres or coverage by hospital budgets. However, in many centres funding is challenging, requires advocacy and time of care providers, and access is only possible after patients fail other immunosuppressants.

Practice point 1.3.3.4 states that low dose alternate day dosing can be prescribed to prevent relapses. This strategy is used by some pediatric nephrology centres across Canada, but not all. The evidence behind this practice is weak as there are no trials comparing outcomes of this strategy versus starting a glucocorticoid-sparing therapy early. This approach is however endorsed as “always” at one Canadian centre, “sometimes” by four centres, and “rarely” used in most centres.

##### Future Directions

Given the multi ancestral backgrounds of Canadian children, the effect of race and ancestry on drug response may need to be taken into consideration in customizing drug protocols. The ancestral differences in incidence and outcomes of childhood NS were explored by Banh et al from data collected through the INSIGHT cohort.^36^ Authors found a higher incidence of disease among South and South East Asian children, but a less complex course compared to Europeans. Similarly, the CHILDNEPH study demonstrated African and Indigenous children received longer courses of corticosteroids compared to white patients.^37^ Building on these clinical observations, recent genome-wide association studies, including a study incorporating patients from the INSIGHT cohort,^38^ have revealed ancestral differences in the genetic risk variants for NS among South Asians, East Asians, and Europeans, particularly at the human leukocyte antigen (HLA) locus.^38-44^ How these genetic variants influence the variable relapsing disease course in NS, both within and across ancestries, and how this genetic information can be implemented in clinical practice is yet to be determined. These considerations are particularly relevant to Canadians who represent multiple ancestries and heritages.

Recent discoveries such as anti-nephrin antibodies^45,46^ and antibodies against other slit diaphragm components^47,48^ in multi-national studies in patients with NS, as well as atypical B cells in children with SSNS from the CHILDNEPH cohort^49^ have demonstrated the crucial role of B cells in the pathophysiology of nephrotic syndrome. Accordingly, many Canadian centres have begun to prioritize the use of Rituximab, a B-cell targeting therapy, in the treatment of FRNS/SDNS. Numerous Canadian centres report having improved access to biosimilars, such as Truxima^TM^ and Ruxience^TM^, rather than to Rituximab itself, which likely means more Canadian sites will be able to use these medications more regularly. There is limited literature detailing the efficacy of biosimilars versus Rituximab in NS, though a recent case series in adults with minimal change disease demonstrated similar effectiveness between agents in inducing remission.^50^ Though the safety profile of biosimilars is similar to Rituximab in other B-cell mediated diseases,^51^ the side effect profile in NS has not been studied. Furthermore, some Canadian centres (2/12) are using new generation B-cell depleting therapies, such as Obinutuzumab and Ofatumumab,^52,53^ in Rituximab-intolerant patients with good effect. Future longitudinal studies characterizing the Canadian experience with new generation B-cell depleting therapies is warranted. Moreover, future efforts to incorporate both anti-nephrin antibodies and atypical B cells as biomarkers for prediction of response to therapy or for prediction of an impending relapse are promising new directions for NS research.

### Treatment of Steroid Resistant Nephrotic Syndrome

#### Recommendation 1.4.1.1

##### Commentary

The definition of steroid resistant nephrotic syndrome (SRNS) in the KDIGO 2025 guidelines is a lack of complete remission after 4 weeks of corticosteroids. The current guidelines also include a confirmation period between 4-6 weeks to determine those that achieve a delayed full remission, labelled as “SSNS late responders”. Fortunately, there is complete harmonization of the SRNS definition between KDIGO and IPNA. The working group is supportive of these definitions in order to properly identify this high-risk population earlier and in a standardized fashion. Recommendation 1.4.4.1 on the use of CNI (cyclosporine or tacrolimus) as first line therapy for SRNS is supported by several studies and is in agreement with the IPNA recommendations. Several previous RCTS, more recent European based registry data (PodoNet), and meta analyses demonstrate that CNI agents are more efficacious in achieving remission and are less likely to cause adverse effects when compared to cyclophosphamide, MMF, chlorambucil, azathioprine and placebo in the treatment of SRNS.^54,55^ Results of genetic testing may further assist in determining response to CNI agents for patients with SRNS.

##### Implications Within Canadian Healthcare

Across Canada, all centres perform a kidney biopsy after establishing steroid resistance; however, resistance has a variable definition across sites. SRNS is defined at 4 weeks in 4/12 centres, 6 weeks at 7/12 centres, and up to 8 weeks at 1/12 centres. Prior to confirming steroid resistance, 3/12 Canadian centres will sometimes trial the use of pulse intravenously-administered methylprednisolone, whereas the remaining 9/12 centres will either rarely or never use this approach. The current guidelines suggest that clinicians may consider a trial of 3 daily doses of intravenous methylprednisolone prior to determining SRNS at 6 weeks. The IPNA guidelines similarly state that SRNS should be defined at 6 weeks, with or without pulse methylprednisolone.^13^ All centres obtain genetic testing in the setting of SRNS, though 3/12 centres report pursuing genetic testing only occasionally. This is likely reflective of the observation that some children with initial SRNS respond to second-line immunosuppression. Indeed, due to delayed access and turnaround time from genetic testing, most Canadian physicians must simultaneously start CNI agents while waiting for genetic testing results to return. This highlights Canadian centres’ reliance on U.S. laboratories for genetic testing services, with the majority (10/12) of Canadian centres obtaining genetic testing from Prevention Genetics, which investigates 74 known genes associated with SRNS. SickKids Hospital in Toronto has an in-house option, but this genetic panel includes only 16 SRNS genes.

Tacrolimus has become the mainstay of CNI treatment in Canada given the relative ease for access, and increased comfort in prescribing given its use in transplant populations. The side effects of cyclosporine, specifically hypertrichosis and gum hypertrophy, can be significantly challenging in the pediatric population. Access to tacrolimus for all children across Canada remains a struggle as some provinces will require special approval or proof of failure of cyclosporine. Children without drug insurance remain specifically at a disadvantage for drug cost coverage. These differences in access to care based on what ultimately reflects socioeconomic status require advocacy and changes at a national level. If children with SRNS do not respond to treatment with CNI, most centres (11/12) will trial treatment with Rituximab, as supported by a recent multi-centred randomized controlled trial.^56^ However, many Canadian centres report funding for Rituximab and time to access to be significant barriers for use. Interestingly, the IPNA guidelines recommend a trial of Rituximab in the setting for CNI-unresponsive SRNS,^13^ whereas the current KDIGO guidelines suggest only a limited role for Rituximab treatment. Future research into the use of anti-slit diaphragm proteins as biomarkers to identify patients with SRNS who are likely to respond to Rituximab is warranted.

#### Future Directions

The use of second-line immunosuppressive medication following steroid resistance is supported by research demonstrating the shared genetic risk between patients with SRNS who test negative on their SRNS gene panel and those who have steroid sensitive disease.^57^ This genetic overlap between gene-test negative NS in all forms (SSNS, SRNS, minimal change disease, and focal segmental glomerulosclerosis) suggests that instead of classifying NS by response to steroids, one could consider classification by immune-mediated versus monogenic cause. Indeed, some Canadian physicians trial CNI agents in SRNS first before pursuing genetic testing. In the setting of response to CNI in SRNS, it may be reasonable to delay genetic testing as a cost-saving measure because those who respond to second-line agents are less likely to have a pathogenic variant in a known SRNS gene. To complicate matters further, a recent multi-centered retrospective study demonstrated that up to 27% of children with a pathogenic variant in a known SRNS gene had partial or complete response to treatment with CNI.^58^ Of course, identification of a pathogenic variant in a known SRNS gene in a patient approaching kidney transplant has the important benefit of counselling for the reduced likelihood of disease recurrence post-transplant.^59^ Further trials examining CNI response in the setting of gene-test negative and monogenic SRNS, and studies to identify CNI response biomarkers are warranted. Generalizability of genetic testing and genetic risk aggregation across populations of different ancestry is also important, as most of the currently published work studies children of White European ancestry only.

## Conclusions

The KDIGO 2025 guidelines offer an important update to the management of children with NS. Its influence is noted in the standardization of aspects of care, notably definitions of disease and response types, induction prednisone protocols and definitions, and treatment of children with SRNS. In this commentary we note the areas that continue to require further standardization such as treatment of NS relapse and selection of second line steroid sparing agents. There have been significant contributions to literature in these areas and it is hoped that nationwide collaborative research will continue to explore and attempt to answer the many remaining questions that exist, allowing practicing physicians to provide evidence based, patient centred care for children with NS.

## Supplemental Material

Supplemental Material - Aligning Practice With Guidelines: A Canadian National Survey and Canadian Society of Nephrology Commentary on the 2025 KDIGO Pediatric Nephrotic Syndrome Recommendations Supplemental Material for Aligning Practice With Guidelines: A Canadian National Survey and Canadian Society of Nephrology Commentary on the 2025 KDIGO Pediatric Nephrotic Syndrome Recommendations by Seetha Radhakrishnan, Allison B. Dart, Mallory L Downie, Silviu Grisaru, Anna Mathew, Reem A. Mustafa and Cherry Mammen in Canadian Journal of Kidney Health and Disease
