# Supplementary material for: Aligning Practice With Guidelines: A Canadian National Survey and Canadian Society of Nephrology Commentary on the 2025 KDIGO Pediatric Nephrotic Syndrome Recommendations
Source: Can J Kidney Health Dis. 2026 May 28;13:20543581261455537. doi: 10.1177/20543581261455537 (PMC13219935; doi:10.1177/20543581261455537)
Supplement: Supplemental Material - Aligning Practice With Guidelines: A Canadian National Survey and Canadian Society of Nephrology Commentary on the 2025 KDIGO Pediatric Nephrotic Syndrome Recommendations [file sj-pdf-1-cjk-10.1177_20543581261455537.pdf]

Appendix A

Working Group Survey: Center Specific Nephrotic Syndrome Practice

Name of hospital/center: [ ]

NS Induction Therapy

For the 1st episode of NS, does your center have a standardized protocol surrounding prednisone treatment?

☐Yes ☐No (If No, consider skipping next parts of the question if practice variation exists at your site).

What dose and duration of prednisone does your center use?

Daily Prednisone Dose

☐60 mg/m^2 ☐2 mg/kg

☐Other: [ ]

Maximum Daily Dose (mg): [ ]

Daily Prednisone Duration

☐6 weeks ☐4 weeks

☐Other [ ]

Alternate Day Prednisone Dose

☐40 mg/m^2 ☐1.5 mg/kg ☐1 mg/kg

☐Other: [ ]

Maximum Alternate Day Dose (mg):

Alternate Day Prednisone Duration

☐6 weeks ☐4 weeks

☐Other:

NS Relapse Therapy

For NS relapses, does your center have a standardized protocol surrounding prednisone treatment?

☐Yes ☐No (If No, consider skipping next parts of the question if practice variation exists at your site).

What dose and duration of prednisone does your center use for NS relapses?

Daily Prednisone Dose

☐60 mg/m^2 ☐2 mg/kg

☐Other:

Maximum Daily Dose (mg):

Daily Prednisone Duration

Alternate Day Prednisone Dose

☐40 mg/m^2 ☐1.5 mg/kg ☐1 mg/kg

☐Other:

Maximum Alternate Day Dose (mg):

Alternate Day Prednisone Duration

☐4 weeks ☐2 weeks

☐Other:

Do you use prolonged courses of low dose, alternate day prednisone for patients with frequently relapsing NS (FRNS) and/or steroid dependent NS (SDNS)?

☐Always ☐Sometimes ☐Rarely ☐Never

Does your center use low dose daily prednisone during infections in to prevent a relapse?

☐Always ☐Sometimes ☐Rarely ☐Never

Steroid Sparing Medications For Frequently Relapsing NS (FRNS) and Steroid Dependent NS (SDNS)

At your center, is there a protocol for selection of initial 2nd line agent/steroid sparing medications for frequently relapsing NS (FRNS) and steroid dependent NS (SDNS) patients?

☐Yes ☐No

Out of the following steroid sparing agents for steroid sensitive patients, please order them from 1-6 in the text box (1 being most commonly prescribed at your site to 6 being least prescribed) for both FRNS & SDNS. If any of the agents are not used/not available, please mark with an "X" beside the treatment. If there are differences in the order/choice between FRNS & SDNS, please specify in the text box.

Cyclophosphamide ☐

MMF ☐

Tacrolimus ☐

Cyclosporine ☐

Rituximab ☐

Levamisole ☐

For the individual steroid sparing medications that are used at your center, please comment on how each of the medications are funded/covered for your patients (eg: provincial funding mechanisms vs private insurance vs hospital based funding for medications like Rituximab)? If funding is a barrier for any of the medications below, please make further comments in the text box

Cyclophosphamide

MMF

Tacrolimus

Cyclosporine

Rituximab

Levamisole

Are there any of the steroid sparing medications you wish you had easier access for your patients and if so, why?

Steroid Resistant NS (SRNS)

Does your center have a standardized definition of steroid resistance in terms of # of weeks of no/partial response?

☐Yes ☐No

If Yes, what is your site's threshold for defining steroid resistance?

☐4 weeks ☐6 weeks ☐8 weeks

☐Other

Do you pursue renal biopsy for patients with SRNS?

☐Yes ☐No ☐Sometimes

Do you pursue genetic testing for patients with SRNS?

☐Yes ☐No ☐Sometimes

How is genetic testing funded at your site?

☐Provincial ☐Hospital ☐Private

☐Other

Does your site utilize IV pulse methylprednisolone when labelled as steroid resistant before starting other therapies?

☐Always ☐Sometimes ☐Rarely ☐Never

Do you use calcineurin inhibitor (CNI) therapy for SRNS?

☐Yes ☐No ☐Sometimes

If Yes/Sometimes, which CNI do you most commonly prescribe at your site for SRNS?

☐Tacrolimus ☐Cyclosporine

Do you use ACE-inhibitors or Angiotensin Receptor Blockers (ARB) for SRNS?

☐Yes ☐No ☐Sometimes

Do you use Rituximab for SRNS patients?

☐Yes ☐No ☐Sometimes

Footnote: Centers who participated in the survey include Alberta Children's Hospital, British Columbia Children's Hospital, Children's Hospital of Eastern Ontario, Children's Hospital London Health Sciences Center, IWK Health Center, Jim Pattison Children's Hospital, McMaster Children's Hospital, Montreal Children's Hospital, Saint Justine Hospital, Stollery Children's Hospital, The Children's Hospital of Winnipeg, and The Hospital for Sick Children
